# Evaluation of the Marburg Heart Score and INTERCHEST score compared to current telephone triage for chest pain in out-of-hours primary care

**DOI:** 10.1007/s12471-022-01745-0

**Published:** 2022-12-29

**Authors:** A. Manten, L. De Clercq, R. P. Rietveld, W. A. M. Lucassen, E. P. Moll van Charante, R. E. Harskamp

**Affiliations:** 1 Department of General Practice, Amsterdam UMC, Amsterdam Cardiovascular Sciences Research Institute, Academic Medical Centre, University of Amsterdam, Amsterdam, The Netherlands; 2 Huisartsenorganisatie Noord-Kennemerland, Alkmaar, The Netherlands; 3 Department of Public and Occupational Health, Amsterdam UMC, Amsterdam Public Health Research Institute, Academic Medical Centre, University of Amsterdam, Amsterdam, The Netherlands

**Keywords:** Chest pain, Primary care, Major events, Acute coronary syndrome

## Abstract

**Introduction:**

Chest pain is a common and challenging symptom for telephone triage in urgent primary care. Existing chest-pain-specific risk scores originally developed for diagnostic purposes may outperform current telephone triage protocols.

**Methods:**

This study involved a retrospective, observational cohort of consecutive patients evaluated for chest pain at a large-scale out-of-hours primary care facility in the Netherlands. We evaluated the performance of the Marburg Heart Score (MHS) and INTERCHEST score as stand-alone triage tools and compared them with the current decision support tool, the Netherlands Triage Standard (NTS). The outcomes of interest were: C‑statistics, calibration and diagnostic accuracy for optimised thresholds with major events as the reference standard. Major events are a composite of all-cause mortality and both cardiovascular and non-cardiovascular urgent underlying conditions occurring within 6 weeks of initial contact.

**Results:**

We included 1433 patients, 57.6% women, with a median age of 55.0 years. Major events occurred in 16.4% (*n* = 235), of which acute coronary syndrome accounted for 6.8% (*n* = 98). For predicting major events, C‑statistics for the MHS and INTERCHEST score were 0.74 (95% confidence interval: 0.70–0.77) and 0.76 (0.73–0.80), respectively. In comparison, the NTS had a C-statistic of 0.66 (0.62–0.69). All had appropriate calibration. Both scores (at threshold ≥ 2) reduced the number of referrals (with lower false-positive rates) and maintained equal safety compared with the NTS.

**Conclusion:**

Diagnostic risk stratification scores for chest pain may also improve telephone triage for major events in out-of-hours primary care, by reducing the number of unnecessary referrals without compromising triage safety. Further validation is warranted.

**Supplementary Information:**

The online version of this article (10.1007/s12471-022-01745-0) contains supplementary material, which is available to authorized users.

## What’s new?

- The Marburg Heart Score and INTERCHEST score have good discriminatory ability and potential for telephone triage of chest pain in out-of-hours primary care.
- Both scores outperform the current triage protocol, and their use would result in a lower number of unnecessary referrals (i.e. false positives) with similar safety (i.e. false negatives) for predicting major events.
- For the prediction of acute coronary syndrome, the INTERCHEST score showed the most favourable discriminatory properties.

## Introduction

Chest pain is a common symptom in out-of-hours primary care (OOH-PC). Only a minority of patients with chest pain suffer from an acute coronary syndrome (ACS) (1.5–3.6%) [1] or another life-threatening disease. Adequate triage is essential to maintain optimal patient flow in the care process and to avoid possible complications associated with life-threatening events. In Dutch primary care, triage is initially through telephone consultation using standardised Netherlands Triage Standard (NTS) protocols [2, 3]. The NTS chest pain protocol was developed based on a consensus of expert opinion and not validated prior to its implementation [4]. A recent regional evaluation revealed that the NTS performs modestly, with a false-positive rate of 83%, and underestimated urgencies in 27% of patients with an ACS or another life-threatening event [5]. Moreover, it was shown that the current triage process leads to many unnecessary referrals, causing an increased workload, overburdening of ambulance services and overcrowding of emergency departments [4, 6].

We postulate that the use of validated clinical decision rules, such as the Marburg Heart Score (MHS) and INTERCHEST score, may also be of added value when employed as a telephone triage tool [7–9]. We therefore set out to evaluate the performance of the MHS and INTERCHEST score as stand-alone triage tools, and to compare their performance with the current NTS protocol on predicting the occurrence of a major event within 6 weeks of initial contact.

## Methods

Our study is reported in accordance with the Standards for Reporting of Diagnostic Accuracy Studies (STARD) 2015 statement (Electronic Supplementary Material, Supplement 5) [10]. The study protocol was approved by our institution’s Medical Ethics Review Committee and registered in the Netherlands Trial Register (TRACE—Trial NL7581) [11].

### Study design

This study was part of the TRiage of Acute Chest pain Evaluation in primary care (TRACE) project. A more elaborate description of the study methods was published previously [12]. In short, this study involved a retrospective, observational cohort of consecutive patients (≥ 18 years) with chest pain who contacted a large, regional primary care facility in Alkmaar, the Netherlands in 2017. This facility provides OOH-PC to approximately a quarter of a million inhabitants. Details on the organisation of Dutch OOH-PC can be found in the Electronic Supplementary Material (Supplement 1).

We gathered registered triage information, including patient and symptom characteristics, urgency levels and course of action following triage, using electronic health records at the OOH-PC facility. Final diagnoses were obtained from the patient file at the affiliated daytime primary care practices, with supporting hospitalisation and/or discharge letters related to the index consultation. Data were collected and processed using a secure, web-based electronic data capture platform (Castor EDC, Amsterdam, The Netherlands) [13].

### NTS triage software

Trained triage assistants handle triage using NTS triage protocols [2]. These protocols are complaint-specific and start with an initial assessment of patients’ vital signs using the ABCDE method [14]. The protocol for chest pain continues with seven questions regarding symptom characteristics (Electronic Supplementary Material, Supplement 1). The NTS algorithm uses the available answers to calculate an urgency level, linked to a recommended time until care. Triage assistants ultimately decide on the most fitting course of action.

### Clinical decision rules

Supplement 2 (Electronic Supplementary Material) gives an overview of the components of each clinical decision rule. The MHS is a point-based score with five elements, developed for ruling out coronary artery disease (CAD) among primary care patients presenting with chest pain [8]. Patients are assigned either 1 (when present) or 0 points (when absent) for each item and the MHS equals the sum, thus ranging from 0 to 5.

The INTERCHEST score is a similar point-based tool developed for the same purpose as the MHS [7]. It consists of six elements, of which five are assigned either 1 (when present) or 0 points (when absent), and one element (‘pain reproducible by palpation’) is given minus 1 point when present and 0 points when absent. Thus, the INTERCHEST score ranges from −1 to +5 points. In order to evaluate the INTERCHEST score as a triage tool, we replaced the physician’s first impression with that of the triage assistant.

### Clinical outcomes: major events

We defined ‘major events’ as a composite of all-cause mortality and urgent conditions, including ACS, linked to the initial complaint of chest pain which required hospital admission and/or urgent in-hospital treatment. Supplement 3 (Electronic Supplementary Material) specifies the diagnoses that constitute major and non-major events. The occurrence of an ACS was based on physician reporting.

### Statistical analysis

We evaluated the performance of the NTS triage software, the MHS and the INTERCHEST score by assessing their discriminative ability (i.e. the ability to distinguish between patients with and without a major event) using C‑statistics and visualised using an area under the receiver operating characteristics curve. We also generated calibration plots to illustrate the agreement between the predicted and observed major event rates. Perfect calibration implies that predictions have an intercept of 0 and a coefficient of 1 (i.e. a diagonal 45-degree slope) [15].

We divided NTS urgency into high (U1–U2; recommended care < 1 h) and low (U3–U5; care > 1 h) and determined the most optimal threshold for the MHS and INTERCHEST score for predicting a major event. When data elements of the MHS or INTERCHEST score were not reported, researchers blinded for clinical outcomes made maximum effort to retrieve the elements from available data. If the data elements were unavailable, they were presumed absent. Patients with an unknown NTS urgency level or without follow-up data were excluded. Hence we did not impute the dataset if triage or outcome data were missing. Furthermore, we performed more restricted analyses specifically for the occurrence of ACS.

## Results

During the study period, a total of 2043 patients reached out to the urgent primary care facility regarding chest pain. We excluded data from 240 patients who opted out of sharing data. We also excluded patients without follow-up information (*n* = 333) or without an NTS software-derived urgency level (*n* = 37), resulting in a final study population of 1433. The characteristics of this population are listed in Tab. 1. Overall, median age was 55.0 years (38.0–71.0), the majority were female (57.6%) and 22.5% had a history of cardiovascular disease.

**Table 1:** Patient characteristics

	Total (*n* = 1433)	Major event (*n* = 235)	Non-major (*n* = 1198)	*p*
Age (IQR)	55.0 (38.0–71.0)	71.0 (61.0–81.0)	51.0 (35.0–68.0)	< 0.001
Sex				
– Male	607 (42.4%)	128 (54.5%)	479 (40.0%)	< 0.001
– Female	826 (57.6%)	107 (45.5%)	719 (60.0%)	< 0.001
Pre-existing medical condition (any)	1061 (74.0%)	209 (88.9%)	852 (71.1%)	< 0.001
History of cardiovascular disease	332 (22.5%)	98 (41.7%)	224 (18.7%)	< 0.001
– Prior CAD^a^	264 (18.4%)	82 (34.9%)	182 (15.2%)	< 0.001
– Prior stroke/TIA	100 (7.0%)	27 (11.5%)	73 (6.1%)	0.003
– Prior peripheral arterial disease	36 (2.5%)	14 (6.0%)	22 (1.8%)	< 0.001
Cardiovascular risk factors (any)	817 (57.0%)	183 (77.9%)	634 (52.9%)	< 0.001
– Smoking (current)	255 (17.8%)	37 (15.7%)	218 (18.2%)	0.37
– Hypertension	446 (31.1%)	121 (51.5%)	325 (27.1%)	< 0.001
– Hypercholesterolaemia	238 (16.6%)	69 (29.4%)	169 (14.1%)	< 0.001
– Diabetes mellitus	176 (12.3%)	54 (23.0%)	122 (10.2%)	< 0.001
Chronic use of medication (any)	917 (64.0%)	192 (81.7%)	725 (60.5%)	< 0.001
– Platelet aggregation inhibitor	91 (6.4%)	28 (11.9%)	63 (5.3%)	< 0.001
– Salicylates	207 (14.4%)	62 (26.4%)	145 (12.1%)	< 0.001
– Vitamin K antagonist	152 (10.6%)	47 (20.0%)	105 (8.8%)	< 0.001
– NOACs	49 (3.4%)	18 (7.7%)	31 (2.6%)	< 0.001
– Beta-blockers	353 (24.6%)	99 (42.1%)	254 (21.2%)	< 0.001
– ACE inhibitors/ARBs	340 (23.7%)	92 (39.1%)	248 (20.7%)	< 0.001
– Lipid-lowering drugs	350 (24.4%)	86 (36.6%)	264 (22.0%)	< 0.001
– Nitrates^b^	149 (10.4%)	52 (22.1%)	97 (8.1%)	< 0.001

The table illustrates the patient characteristics for both the total group of patients (*n* = 1433) and after division based on the occurrence of a major event. Continuous variables are presented as medians (IQR) due to a non-normal distribution

*IQR* interquartile range, *CAD* coronary artery disease, *TIA* transient ischaemic attack, *NOAC* novel oral anticoagulant, *ACE* angiotensin-converting enzyme, *ARB* angiotensin receptor blocker

^a^Defined as a history of both acute and stable coronary syndromes

^b^Both chronic and PRN (pro re nata) use of nitrates

Major events occurred in 16.4% (*n* = 235). Cardiovascular conditions, such as ACS, comprised the majority of the major events (181/235, 77.0%), followed by respiratory and abdominal aetiologies (9.8 and 7.2%, respectively) (Electronic Supplementary Material, Supplement 4). Overall, patients with a major event were older, more often male and more likely to have pre-existing medical conditions.

### Performance of the NTS

**Fig. 1:**
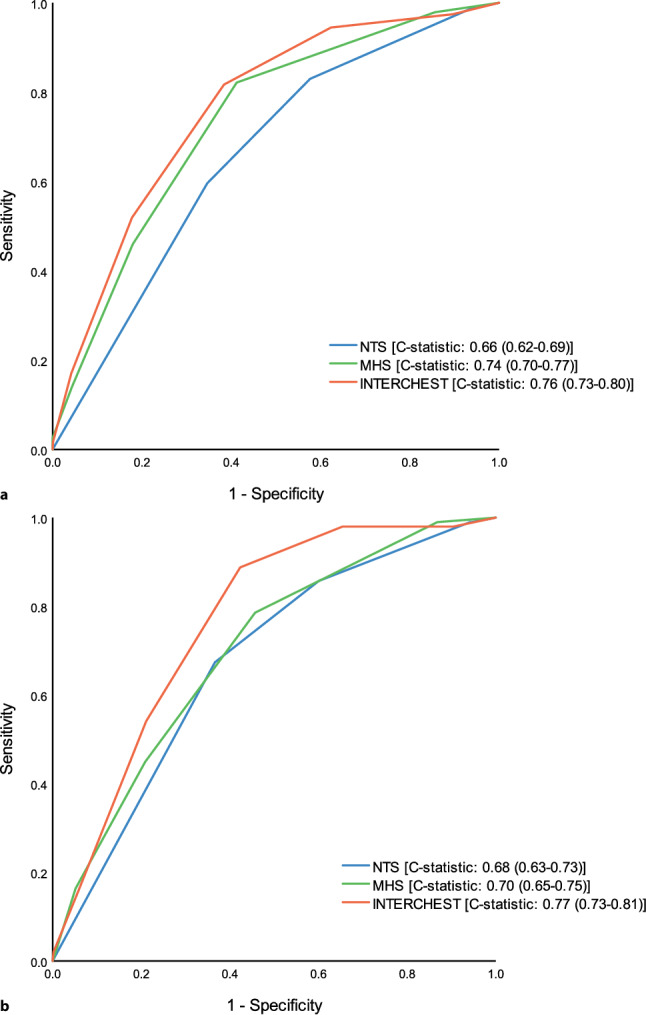
AUROC (area under the receiver operating characteristic) curves for major events (**a**) and acute coronary syndrome (ACS, **b**). The curves were based on the assigned urgency level (Netherlands Triage Standard, *NTS*) and calculated risk scores (Marburg Heart Score [*MHS*] and INTERCHEST score) regarding the occurrence of a major event or ACS

**Fig. 2:**
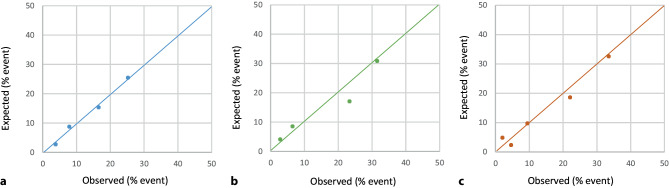
Calibration plots of the NTS software, Marburg Heart Score and INTERCHEST score for major events. **a** Calibration plot of NTS software, **b** Calibration plot of Marburg Heart Score, **c** Calibration plot of INTERCHEST score

Performance analyses of the NTS triage software were based on the urgency level allocation and the occurrence of a major event. Fig. 1 illustrates the discriminative properties with a C-statistic of 0.66 (95% confidence interval [CI]: 0.62–0.69). Calibration was adequate when comparing observed versus expected major events (Fig. 2). Diagnostic properties were calculated after dividing urgencies into high (U1–U2) and low (U3–U5) (Tab. 2), resulting in 886 (61.8%) recommended referrals (i.e. high urgency), of which 691 (48.2%) were unnecessary (i.e. false positive). In this case, 40 of 235 (17.0%) major events would have been missed (i.e. false negative). The sensitivity and specificity of the NTS were 83.0 and 42.3%, respectively.

**Table 2:** Diagnostic properties of Netherlands Triage Standard (*NTS*), Marburg Heart Score (*MHS*) and INTERCHEST score at different thresholds for predicting major events

	Threshold (score)	TP	FN	FP	TN	Sensitivity (%)	Specificity (%)	PPV (%)	NPV (%)	Accuracy (%)
*NTS*	U1/U2	195	40	691	507	83.0 (77.6–87.6)	42.3 (39.5–45.2)	22.0 (20.7–23.3)	92.7 (90.5–94.4)	49.0 (46.4–51.6)
*MHS*	≥ 1	230	5	1026	172	97.9 (95.1–99.3)	14.4 (12.4–16.5)	18.3 (17.9–18.8)	97.2 (93.5–98.8)	28.1 (25.7–30.5)
	≥ 2	193	42	494	704	82.1 (76.6–86.8)	58.8 (55.9–61.6)	28.1 (26.3–30.0)	94.4 (92.7–95.7)	62.6 (60.0–65.1)
*INTERCHEST*	≥ 1	222	14	747	451	94.1 (90.3–96.7)	37.7 (34.9–40.5)	22.9 (22.0–23.9)	97.0 (95.1–98.2)	46.9 (44.3–49.6)
	≥ 2	192	43	460	738	81.7 (76.2–86.4)	61.6 (58.8–64.4)	29.5 (27.5–31.4)	94.5 (92.9–95.8)	64.9 (62.4–67.4)

The table shows thresholds only for the MHS and INTERCHEST score with at least similar false-negative rates for major events. Test properties are displayed as percentages with the 95% confidence interval in parentheses

*TP* true positive, *FN* false negative, *FP* false positive, *TN* true negative, *PPV* positive predictive value, *NPV* negative predictive value

### Performance of the MHS

The MHS produced a C-statistic of 0.74 (95% CI: 0.70–0.77) (Fig. 1) and calibration appeared adequate (Fig. 2). Diagnostic properties were calculated at different thresholds. A threshold of 2 points showed optimal diagnostic accuracy with a sensitivity of 82.1% and a specificity of 58.8% (Tab. 2). Compared to the NTS software, MHS ≥ 2 resulted in 199 fewer referrals (−22.5%) and only two additional missed major events.

### Performance of the INTERCHEST score

The INTERCHEST score resulted in a C-statistic of 0.76 (95% CI: 0.73–0.80) (Fig. 1), and calibration appeared to be reasonably good (Fig. 2). An INTERCHEST score with a threshold of ≥ 2 points showed optimal diagnostic accuracy with a sensitivity and specificity of 81.7 and 61.6%, respectively. Compared to the NTS, the INTERCHEST score improved efficiency by leading to fewer patient referrals (−234) with a similar number of missed cases (*n* = 43 vs *n* = 40).

### Performance of NTS, MHS and INTERCHEST score regarding ACS

Overall, 98 (6.8%) patients suffered from an ACS. For the occurrence of an ACS, C‑statistics were 0.68 (95% CI: 0.63–0.73), 0.70 (0.65–0.75) and 0.77 (0.73–0.81) for the NTS, MHS and INTERCHEST score, respectively (Fig. 1). The NTS software showed a sensitivity of 85.7% and specificity of 39.9%, with 14 missed cases and an unnecessary referral rate of 56% (*n* = 802) (Tab. 3). The MHS did not outperform the NTS, with more missed cases (*n* = 7) and a sensitivity and specificity of 78.6 and 54.3%, respectively. The INTERCHEST score showed more favourable diagnostic capability, with a sensitivity and specificity of 88.8 and 57.6%, 234 fewer referrals and fewer missed ACS diagnoses (*n* = −3).

**Table 3:** Diagnostic properties of the Netherlands Triage Standard (*NTS*), Marburg Heart Score (*MHS*) ≥ 2 and INTERCHEST score ≥ 2 for acute coronary syndrome

	Threshold (score)	TP	FN	FP	TN	Sensitivity (%)	Specificity (%)	PPV (%)	NPV (%)	Accuracy (%)
*NTS*	U1/U2	84	14	802	533	85.7 (77.2–92.0)	39.9 (37.3–42.6)	9.5 (8.7–10.3)	97.4 (95.9–98.4)	43.1 (40.5–45.7)
*MHS*	≥ 2	77	21	610	725	78.6 (69.1–86.2)	54.3 (51.6–57.0)	11.2 (10.1–12.5)	97.2 (95.9–98.1)	56.0 (53.4–58.6)
*INTERCHEST*	≥ 2	87	11	565	770	88.8 (80.8–94.3)	57.7 (55.0–60.4)	13.3 (12.3–14.5)	98.6 (97.6–99.2)	59.8 (57.2–62.4)

Test properties are displayed as percentages with the 95% confidence interval in parentheses

*TP* true positive, *FN* false negative, *FP* false positive, *TN* true negative, *PPV* positive predictive value, *NPV* negative predictive value

## Discussion

Adequate classification of patients with chest pain is paramount for any given triage system, certainly when an unselected patient population is involved. Balancing maximum efficiency while minimising the number of underestimated cases is a daunting challenge. Our study evaluated the use of existing chest-pain-specific clinical decision rules (MHS and INTERCHEST score) as stand-alone triage tools, and compared these to the current triage protocol (NTS). We found that the diagnostic capability of the NTS is modest at best for discriminating between patients with and without a major event or ACS. Both the MHS (≥ 2) and the INTERCHEST score (≥ 2) outperformed the NTS by improving efficiency without negatively impacting on false-negative rates.

### Strengths and limitations

To our knowledge, this is the first study to evaluate and compare the test accuracy of an existing telephone triage tool with validated diagnostic prediction rules in OOH-PC for a broader set of chest-pain-related conditions. We assessed a relatively large study population, comprising all chest pain patients that contacted OOH-PC without further selection. While the organisational structures for European OOH-PC show several differences between and within countries, general practitioners (GPs) are generally positioned as gate-keepers to secondary care and often use integrated telephone triage systems [3]. Our findings may therefore be generalisable to similar settings across Europe.

The main limitation of our study is its retrospective nature. Verification bias may have been in play, since only patients in whom there was a high suspicion of a major event underwent further diagnostic work-up to confirm or rule out the occurrence of an event. However, we tried to minimise this bias by using a 6-week follow-up window for the outcome of interest, allowing us to capture initially missed events. Second, since the MHS and INTERCHEST score are not integrated into current practice, score elements were not registered structurally. We presumed the absence of a symptom or score element when it was not recorded by the triage assistant. Third, a mentionable amount of lost-to-follow-up was caused by GPs refusing to provide data on their patients (6.0% of baseline patients). These GPs expressed concerns due to the recent implementation of the European General Data Protection Regulation.

### Prior research on the MHS and INTERCHEST score

The MHS was developed by Bösner et al. [8] for predicting CAD in daytime primary care among patients with chest pain. Overall, best results were obtained when applying a threshold of 3 points, and repeated validation showed a discriminative ability ranging from 0.84 to 0.90, with good sensitivity (87–91%) and specificity (61–81%) [8, 9, 16]. Additional external validation of the MHS among patients in Southeast Asia resulted in a less favourable discriminative ability of 0.66 (95% CI: 0.62–0.70) [17]. The MHS was also tested for its ability to rule out ACS among a selected patient population referred for immediate cardiac evaluation because an ACS was suspected [18]. Results of this study showed that the MHS alone had insufficient diagnostic accuracy (75.0% sensitivity and 44.0% specificity), and incorporation of the MHS in GP consultations produced a specificity of only 23.3% despite an increased sensitivity (94.4%). In the present study, the MHS was evaluated as a stand-alone triage tool for distinguishing between patients with and without major events. While the rule was not constructed for this purpose, it still outperformed the NTS with C‑statistics of 0.74 versus 0.66 and increased specificity (+16.5%).

Similar to the MHS, the INTERCHEST score was designed for ruling out CAD in daytime primary care [7]. It was based on five cohort studies, including the MHS derivation study. Overall, studies found a sensitivity of 82–88% and specificity of 74–82% for ruling out CAD (threshold ≥ 2) [16]. In urgent primary care, the INTERCHEST score demonstrated good discriminative ability (C-statistic > 0.80) for predicting major adverse cardiovascular events [19]. The usability of the INTERCHEST score seems to be corroborated by the present study, which showed it to have the most favourable diagnostic capability for predicting both major events and ACS.

### Implications for future research

Our results illustrate how existing clinical decision rules may improve triage risk-stratification in OOH-PC settings. However, in the light of the retrospective nature of our study, we should first conduct further prospective validation, preferably at multiple sites and settings, before recommending implementation in routine care. We believe that such studies should not focus on ACS alone, but rather focus on a broader set of chest-pain-related outcomes (or major events) that are relevant in unselected patient populations.

## Conclusion

The Marburg Heart Score and INTERCHEST score have good discriminative ability for telephone triage purposes in primary care patients with chest pain. Both scores outperformed the current triage protocol in our study, but further prospective validation is warranted.

## Supplementary Information

**Supplement 1. **Text box explaining the organizational structure of Dutch out-of-hours primary care

**Supplement 2.** Components of the triage protocol and the two clinical decision rules

**Supplement 3.** Definition of major and non-major events

**Supplement 4.** Subdivision of final diagnoses and their frequencies among the total group of patients and among the patients who suffered a major event

**Supplement 5.** STARD
