# Supplementary material for: Evaluation of the Marburg Heart Score and INTERCHEST score compared to current telephone triage for chest pain in out-of-hours primary care
Source: Neth Heart J. 2022 Dec 29;31(4):157–65. doi: 10.1007/s12471-022-01745-0 (PMC10033786; doi:10.1007/s12471-022-01745-0)
Supplement: Supplementary file 1 — Supplement 1. Text box explaining the organizational structure of Dutch out-of-hours primary care [file 12471_2022_1745_MOESM1_ESM.docx]

## **Supplement 1.** Text box explaining the organizational structure of Dutch out-of-hours primary care

**Organizational structure of out-of-hours primary care in the Netherlands**

The dominant organizational structure for Dutch out-of-hours primary care is formed by general practitioner (GP) cooperatives. These large-scale, regional facilities provide care to the patients of the affiliated GPs during evenings, weekends and national holidays. Upon contact, patients are triaged in order to assess the urgency of their complaint. In most Dutch out-of-hours primary care facilities, trained triage assistants handle triage with the help of standardized protocols from the “Netherlands Triage Standard” (NTS). These protocols are complaint-specific and developed using 1) expert opinion, 2) a modified version of the Manchester Triage System, and 3) existing telephone guidelines used in primary care. However. The resulting protocols were never validated before their implementation in 2011 and the exact algorithm behind the NTS protocols remains unknown to the public.

All of the NTS protocols start with a check of vital signs using the ABCDE-method, followed by a number of hierarchically structured questions. The specific protocol for evaluating chest pain consists of seven questions encompassing the type, duration, severity, course of the pain, location on the chest, presence of radiation, and associated symptoms (i.e. diaphoresis, nausea, pallor, anxiety, fainting). The NTS algorithm continuously calculates a recommended urgency level based on the responses (minimum of 1). As shown in the table below, each urgency level is linked to a recommended time-until-care. Additional information from the triage conversation (e.g. additional symptoms or information from para-language) can be registered by the triage assistant in a free text box and is not included in the algorithm’s calculations. However, triage assistants are able to overrule the NTS urgency if deemed necessary and ultimately decide on the most fitting course of action (i.e. ambulance activation, face-to-face GP consultation, referral to the own GP during office-hours or self-care advice).


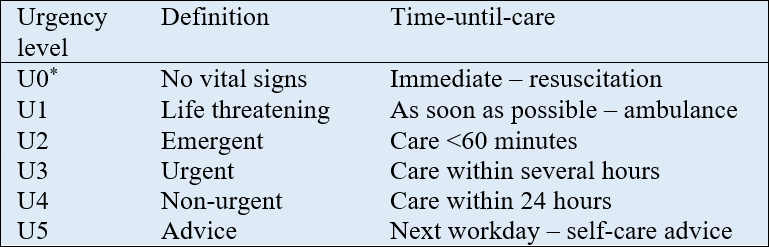


*Notes:* ^*^U0 urgency levels are reserved to patients in need of resuscitation, in such cases triage is not initiated and an ambulance is immediately activated.
