# Supplementary material for: Evaluation of the Marburg Heart Score and INTERCHEST score compared to current telephone triage for chest pain in out-of-hours primary care
Source: Neth Heart J. 2022 Dec 29;31(4):157–65. doi: 10.1007/s12471-022-01745-0 (PMC10033786; doi:10.1007/s12471-022-01745-0)
Supplement: Supplementary file 2 — Supplement 2. Components of the triage protocol and the two clinical decision rules [file 12471_2022_1745_MOESM2_ESM.docx]

## **Supplement 2.** Components of the triage protocol and the two clinical decision rules

| **Netherlands Triage Standard** | **Marburg Heart Score** | **INTERCHEST Score** |
| --- | --- | --- |
| Check of vital signs | Age/sex (F ≥65 years, M ≥55 years) | Age/sex (F ≥65 years, M ≥55 years) |
| Type of pain | Known CVD^*^ | History of CAD^†^ |
| Duration of the pain | Pain worse with exercise | Chest pain related to effort |
| Severity | Pain not reproducible by palpation | Pain reproducible by palpation |
| Course of the pain | Patient assumes pain is of cardiac origin | Triage assistant suspects serious condition^‡^ |
| Location on the chest |  |  |
| Radiation |  | Chest pain feels like “pressure” |
| Associated symptoms |  |  |
| *Range: U1 – U5 level* | *Range: 0 – 5 points* | *Range: -1 – 5 points* |

*Supplement 2. Table with the components of the triage protocol and the two clinical decision rules.*
*Abbreviations:* F = female, M = male, CVD = clinical vascular disease, CAD = coronary artery disease *Notes:* ^*^ History of myocardial infarction, transient ischemic attack, cerebrovascular accident, peripheral artery disease, previous percutaneous coronary intervention or coronary artery bypass graft. ^†^ History of myocardial infarction, previous percutaneous coronary intervention or coronary artery bypass graft. ^‡^ In order to evaluate the INTERCHEST score in a triage setting, we replaced the physician’s suspicion of a serious condition with that of the triage assistant. The component ‘suspicion’ was based on the triage assistants reaction based on their first impression or ‘gut feeling’. For instance, this item was considered present when patients received immediate ambulance activation, when triage assistants explicitly noted their concern or when assistants discussed the case with the attending GP due to doubt.
