# Supplementary material for: Evaluation of the Marburg Heart Score and INTERCHEST score compared to current telephone triage for chest pain in out-of-hours primary care
Source: Neth Heart J. 2022 Dec 29;31(4):157–65. doi: 10.1007/s12471-022-01745-0 (PMC10033786; doi:10.1007/s12471-022-01745-0)
Supplement: Supplementary file 3 — Supplement 3. Definition of major and non-major events [file 12471_2022_1745_MOESM3_ESM.docx]

## **Supplement 3.** Definition of major and non-major events

|  | **Final diagnosis** | **Conditional management** |
| --- | --- | --- |
| **Major event** | Death from any cause |  |
|  | Acute coronary syndrome |  |
|  | Urgent coronary revascularization |  |
|  | Pulmonary embolism |  |
|  | Thoracic aortic aneurysm (dissection or ruptured) |  |
|  | Severe/Acute congestive heart failure | Hospitalization |
|  | Severe peri(myo)carditis | Hospitalization |
|  | Symptomatic atrial fibrillation | Hospitalization (cardioversion or converted through medication) |
|  | Aortic valve stenosis | Hospitalization |
|  | (Tension) pneumothorax | Hospitalization |
|  | Severe pneumonia | Hospitalization |
|  | CVA / TIA |  |
|  | Inflammatory processes such as appendicitis, pancreatitis, cholecystitis | Hospitalization |
|  | Other, such as: exacerbation COPD or hypertensive crisis | Hospitalization |
|  | Traumatic event (with significant impact) | Hospitalization |
| **Non-major event** | Stable angina pectoris | Outpatient treatment |
|  | Mild congestive heart failure | Outpatient treatment |
|  | Mild peri(myo)carditis | Outpatient treatment |
|  | Atrial fibrillation (recurrent / paroxysmal) | Outpatient treatment |
|  | Hypertension | Outpatient treatment |
|  | Mild pneumothorax | Outpatient treatment |
|  | Mild pneumonia | Outpatient treatment |
|  | Mild respiratory problems (such as viral infections) |  |
|  | Gastric/oesophagus problems |  |
|  | Muscoloskeletal |  |
|  | Traumatic (mild trauma) | Outpatient treatment |
|  | Mental health / Panic attack / Anxiety disorder |  |

*Supplement 3. Definition of major and non-major events*
Major event was defined as a composite of all-cause mortality and urgent medical conditions linked to the initial complaint of chest pain which required hospital admittance, and/or urgent in-hospital treatment. Thus, major events includes both cardiovascular, as well as non-cardiovascular conditions. The table specifies the subdivision between major and non-major events, and states the conditional managements for some of these diagnoses in the right column.
*Abbreviations:* cerebrovascular accident (CVA), transient ischaemic attack (TIA), chronic obstructive pulmonary disease (COPD).
