# Supplementary material for: Evaluation of the Marburg Heart Score and INTERCHEST score compared to current telephone triage for chest pain in out-of-hours primary care
Source: Neth Heart J. 2022 Dec 29;31(4):157–65. doi: 10.1007/s12471-022-01745-0 (PMC10033786; doi:10.1007/s12471-022-01745-0)
Supplement: Supplementary file 4 — Supplement 4. Subdivision of final diagnoses and their frequencies among the total group of patients and among the patients who suffered a major event [file 12471_2022_1745_MOESM4_ESM.docx]

## **Supplement 4.** Subdivision of final diagnoses and their frequencies among the total group of patients and among the patients who suffered a major event.

##

|  | | | | **Total**  (n=1,433) | **Major events**  (n=235) |
| --- | --- | --- | --- | --- | --- |
| **Cardiovascular** | | | | **359 (25.1%)** | **181 (77.0%)** |
|  | Acute coronary syndrome | | | 98 (6.8%) | 98 (41.7%) |
|  | Chronic coronary syndrome *(e.g. stable angina, ischaemic disease without angina)* | | | 68 (4.7%) | 8 (3.4%) |
|  | Atrial fibrillation | | | 61 (4.3%) | 38 (16.2%) |
|  | Heart failure | | |  |  |
|  |  | Congestive heart failure^*^ | | 22 (1.5%) | 13 (5.5%) |
|  |  | Other *(including cardiomyopathy)* | | 2 (0.1%) | - |
|  | Peri(myo)carditis | | | 16 (1.1%) | 3 (1.3%) |
|  | Aortic aneurysm or dissection | | | 3 (0.2%) | 3 (1.3%) |
|  | Pulmonary embolism | | | 12 (0.8%) | 9 (3.8%) |
|  | Cerebrovascular disease *(CVA and TIA)* | | | 2 (0.1%) | 2 (0.9%) |
|  | Abnormal blood pressure | | |  |  |
|  |  | Hypertension | | 16 (1.1%) | 1 (0.4%) |
|  |  | Postural hypotension | | 3 (0.2%) | - |
|  | Arrhythmias and conduction disorders | | |  |  |
|  |  | Tachycardias *(paroxysmal, SVT, VT)* | | 6 (0.4%) | 2 (0.9%) |
|  |  | Other *(extrasystole, atrioventricular block, presence of cardiac device)* | | 6 (0.4%) | - |
|  | Valvular disorders | | | 4 (0.3%) | 1 (0.4%) |
|  | Other cardiovascular  *(e.g. palpitations NOS, heartdisease NOS)* | | | 40 (2.8%) | 3 (1.3%) |
| **Musculoskeletal** | | | **652 (45.5%)** | | **8 (3.4%)** |
|  | Chest wall pain *(including herpes zoster, Tietze and breast pain)*^†^ | | | 578 (40.3%) | 5 (2.1%) |
|  | Musculoskeletal other | | |  |  |
|  |  | Neck and back pain/complaints | | 15 (1.0%) | - |
|  |  | Arm and shoulder pain/complaints | | 16 (1.1%) | - |
|  |  | Traumatic *(e.g. traumatic ruptured spleen, contusion or fractured ribs)* | | 32 (2.2%) | 3 (1.3%) |
|  |  | Unspecified *(e.g. muscle pain, gout)* | | 11 (0.8%) | - |
| **Respiratory** | | | | **122 (8.5%)** | **23 (9.8%)** |
|  | Pneumonia | | | 38 (2.7%) | 15 (6.4%) |
|  | Pneumothorax | | | 3 (0.2%) | 2 (0.9%) |
|  | Other respiratory | | |  |  |
|  |  | Infectious other *(e.g. upper respiratory tract infections, viral pleurisy, bronchiolitis)* | | 27 (1.9%) | - |
|  |  | Chronic pulmonary diseases *(e.g. asthma, COPD)* | | 22 (1.5%) | 3 (1.3%) |
|  |  | Respiratory complaints other *(including cough, dyspnoea, pain, known malignancy)* | | 32 (2.2%) | 3 (1.3%) |
| **Abdominal** | | | | **152 (10.6%)** | **17 (7.2%)** |
|  | Stomach and oesophageal related complaints/diseases *(e.g. heartburn, reflux disease, nausea, indigestion, epigastric pain)* | | | 82 (5.7%) | 2 (0.9%) |
|  | Biliary diseases *(cholelithiasis, cholecystitis)* | | | 21 (1.5%) | 5 (2.1%) |
|  | Pancreatitis | | | 3 (0.2%) | 2 (0.9%) |
|  | Abdominal other | | |  |  |
|  |  | Undifferentiated abdominal pain | | 12 (0.8%) | - |
|  |  | Inflammatory *(appendicitis, chronic enteritis)* | | 3 (0.2%) | 2 (0.9%) |
|  |  | Urological *(urinary infection, urinary calculus)* | | 14 (1.0%) | 4 (1.7%) |
|  |  | Other unspecified *(including diverticular disease, constipation)* | | 17 (1.2%) | 2 (0.9%) |
| **Psychological and mental health** | | | | **95 (6.6%)** | **1 (0.4%)** |
|  | Anxiousness / fear of disease / panic disorders  *(including hyperventilation)* | | | 82 (5.7%) | - |
|  | Other psychological and mental health | | |  |  |
|  |  | Substance abuse and intoxications^‡^ | | 5 (0.3%) | 1 (0.4%) |
|  |  | Other unspecified *(including dementia, delirium)* | | 8 (0.6%) | - |
| **Other** | | | | **53 (3.7%)** | **5 (2.1%)** |
|  | General and unspecified diagnosis *(e.g. pain, fever, weakness, allergies and adverse drug reactions)* | | | 22 (1.5%) | 3 (1.3%) |
|  | Neurological *(including headache, dizziness, fainting and sensory complaints)* | | | 21 (1.5%) | - |
|  | Skin/tissue problems | | | 3 (0.2%) | - |
|  | Endocrine system  *(including thyroid problems and diabetes)* | | | 4 (0.3%) | 2 (0.9%) |
|  | Haematological | | | 3 (0.2%) | - |

*Supplement 3. Table illustrating the subdivision of final diagnoses and their frequencies among the total group of patients and among the patients who suffered a major event.*
*Abbreviations:* Cerebrovascular accident (CVA), transient ischemic attack (TIA), supraventricular tachycardia (SVT), ventricular tachycardia (VT), not otherwise specified (NOS), chronic obstructive pulmonary disease (COPD).
*Notes:* ^*^ Includes 1 patient witch cardiac asthma without ischemia. ^†^ Includes one patient with chest pain due to progressive Non-Hodgkin’s lymphoma, the progression caused an impeding spinal cord injury. ^‡^ Specifically no cases of cocaine induced ischemia.
